# MBTPS2, a membrane bound protease, underlying several distinct skin and bone disorders

**DOI:** 10.1186/s12967-021-02779-5

**Published:** 2021-03-20

**Authors:** Natarin Caengprasath, Thanakorn Theerapanon, Thantrira Porntaveetus, Vorasuk Shotelersuk

**Affiliations:** 1 Center of Excellence for Medical Genomics, Medical Genomics Cluster, Department of Pediatrics, Faculty of Medicine, Chulalongkorn University, Bangkok, 10330 Thailand; 2 Excellence Center for Genomics and Precision Medicine, King Chulalongkorn Memorial Hospital, The Thai Red Cross Society, Bangkok, 10330 Thailand; 3 Genomics and Precision Dentistry Research Unit, Department of Physiology, Faculty of Dentistry, Chulalongkorn University, Bangkok, 10330 Thailand

**Keywords:** BRESHECK, KFSD, IFAP, Olmsted syndrome, Osteogenesis imperfecta, S2P

## Abstract

The *MBTPS2* gene on the X-chromosome encodes the membrane-bound transcription factor protease, site-2 (MBTPS2) or site-2 protease (S2P) which cleaves and activates several signaling and regulatory proteins from the membrane. The MBTPS2 is critical for a myriad of cellular processes, ranging from the regulation of cholesterol homeostasis to unfolded protein responses. While its functional role has become much clearer in the recent years, how mutations in the *MBTPS2* gene lead to several human disorders with different phenotypes including Ichthyosis Follicularis, Atrichia and Photophobia syndrome (IFAP) with or without BRESHECK syndrome, Keratosis Follicularis Spinulosa Decalvans (KFSD), Olmsted syndrome, and Osteogenesis Imperfecta type XIX remains obscure. This review presents the biological role of MBTPS2 in development, summarizes its mutations and implicated disorders, and discusses outstanding unanswered questions.

## Introduction

The Membrane-Bound Transcription factor Protease, Site-2 gene (*MBTPS2*) on the X-chromosome encodes site-2 protease (S2P) which is an integral membrane protein that plays vital roles in regulating membrane-tethered transcriptional factors. It is ubiquitously expressed within the Golgi membrane where it functions sequentially with site-1 protease (S1P), encoded by the membrane-bound transcription factor protease, site-1 (MBTPS1), to proteolytically activate membrane-tethered latent transcription factors. This mechanism is known as regulated intramembrane proteolysis (RIP).

RIP is a tightly organized process that is critical for cellular signal transductions and the regulation of diverse processes, such as cellular division and differentiation, cell migration, transcriptional regulation, apoptosis, cellular stress responses, degradation of transmembrane protein fragments, and lipid metabolism as well as a plethora of physiological processes including embryonic development and normal functioning of the nervous and immune system [1]. Disruption or deregulation of RIP have been implicated in the pathogenesis of several diseases such as cancer, Alzheimer’s disease, and developmental disorders [2–4].

**Fig. 1:**
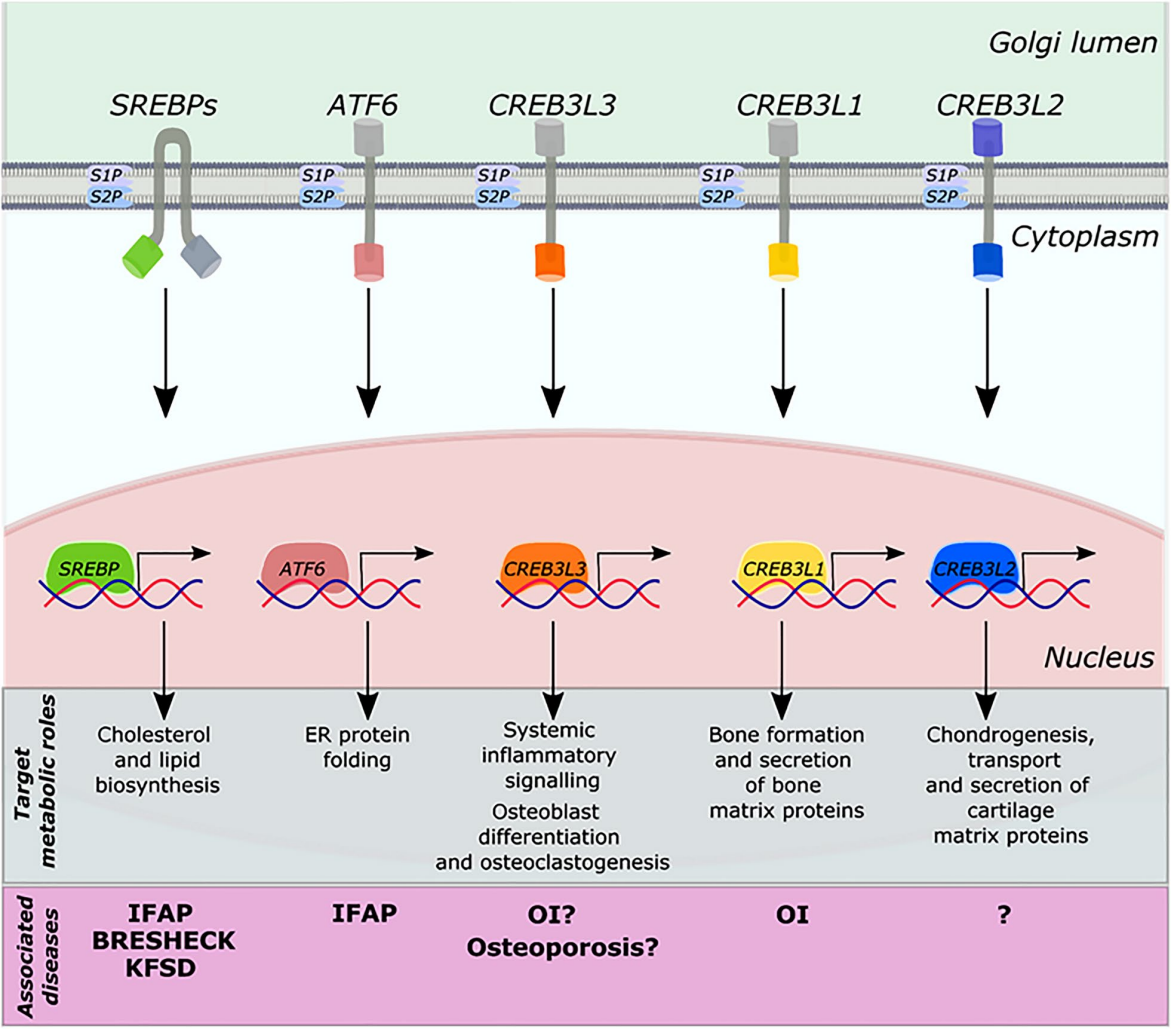
Schematic illustration of S1P and S2P mediated RIP. Following stimulation, substrates are translocated from the ER to Golgi. At the Golgi, they are cleaved by S1P and then by S2P, liberating their N-terminal domains from the membrane to activate genes in the nucleus and thereby initiating signaling pathways involved in lipid metabolism, protein folding, osteogenesis, inflammatory signaling, osteoclastogenesis, chondrogenesis, and secretion of extracellular matrix proteins. Impairments of S2P lead to IFAP syndrome with or without BRESHECK, KFSD, OS and OI

RIP generally employs a two-step sequential cleavage process (Fig. 1). The first cleavage, either constitutively or in response to cell stimuli, cleaves a membrane protein substrate close to or within its transmembrane (TM), resulting in the release of the soluble extracellular domain (ectodomain) from the membrane and a fragment containing one or more functional domains remains bound to the membrane [5]. The remaining membrane-embedded fragment is then recognized by a second protease and is cleaved within the TM region, liberating the fragment into the cytoplasm. Following its release, the fragment is either translocated to the nucleus where it functions as a transcription regulator [2] or acts as an activator of different signaling pathways [6].

The second intramembrane cut, that releases the membrane bound fragments into the cytosol is carried out by one of the four distinct and evolutionarily conserved proteases named intramembrane cleaving proteases (I-CLiPs): the aspartyl protease-like, the zinc metalloproteinase S2P, the serine protease family of rhomboids, and the glutamatergic intramembrane proteases [7]. Among the four I-CLiPs, the S2P was the first to be discovered with a role involved in the feedback regulation of sterol and fatty acid biosynthesis and uptake by controlling the activity of the membrane-bound transcription factors sterol regulator element binding proteins (SREBPs) [8–10]. Subsequently, additional transcriptional factors were identified for other pathways such as activating transcription factor 6 (ATF6) [11] and cyclic AMP-responsive element-binding protein 3-like protein 3 (CREB3L3) [12]. Of late, newer roles have been attributed to *MBTPS2* (MIM #300,294), not only for the transcription factors that it processes but also for its involvement in unexpected pathways that are critical for diverse biological process, such as ER stress, unfolded protein response, and gluconeogenesis [13]. Accordingly, MBTPS2 contributes to the pathogenesis of several X-linked disorders.

In this review, we focus on MBTPS2 and provide an update overview of the current understanding of how MBTPS2 contributes to a range of functions in maintaining cellular integrity and examine diseases caused by *MBTPS2* mutations. We highlight the role of MBTPS2 in skin disorders and X-linked recessive form of osteogenesis imperfecta (X-OI), as these pathological states provide good examples of the diversified functions and their dysfunction in diseases.

## Substrates cleaved by S2P

### Discovery of S2P and the identification of SREBPs as its first substrates

The landmark discovery of the human zinc metalloproteinase S2P in 1997 as a critical component of the processing machinery in cleaving TM precursor proteins has transformed our conceptual molecular understanding of the regulation of fundamental cellular processes carried out via RIP. Somewhat serendipitous, S2P was discovered through a series of independent studies that deciphered the SREBP pathway to understand the global regulation of cholesterol homeostasis in mammalian cells [8, 10]. In fact, the requirement of proteolysis of SREBPs (SREBP-1 and SREBP-2) to maintain cholesterol homeostasis was well-understood prior to the discovery of S2P [14]. However, it was not until the seminal observation that for SREBPs to fulfill its role in cholesterol homeostasis, they must undergo a two-step proteolytic cascade, owing to the distinctive protein structure of the SREBPs [8].

Dissimilar to other transcriptional factors, SREBPs are translated as inactive precursors anchored to the ER membrane. The SREBP precursor proteins, approximately 1,150 amino acids in length, are composed of three domains. The amino (NH_2_)-terminal DNA-binding domain is a basic-helix-loop-helix-leucine zipper (bHLHL-Zip) family of transcription factors. The middle domain is a helical hairpin membrane anchor, with two TM helices separated by a short loop that projects into the lumen of the ER and nuclear envelope. The carboxy (COOH)-terminal, which also projects in the cytosol, functions as the regulatory domain [15, 16]. In response to sterol deprivation, precursor SREBPs are transported from the ER membrane to the Golgi where it is cleaved by two site-specific proteases. The first protease cleaves the precursor SREBP proteins at site 1, a conserved leucine residue within the luminal loop, separating the SREBPs into two halves but the bHLHL-Zip domain remains bound to the membrane. Following this, a second protease, cleaves the NH_2_-terminal intermediate domain at site 2, a site within the transmembrane helix, to release the bHLHL-Zip domain from the membrane. The bHLHL-Zip domain is then translocated to the nucleus to initiate the transcription of genes encoding enzymes involved in the biosynthesis and uptake of cholesterol, fatty acids, and triglyceride (Fig. 1). The initial proteolytic cleavage is regulated by sterol levels whereas the cleavage occurring at site 2 is not, however, cleavage at site 2 can only occur following the site 1 cleavage [8].

The characterization and appreciation of the vital role that these cleavage machineries serve in the feedback regulatory system of cholesterol synthesis and uptake warranted studies to identify these proteases. Grasping this opportunity was the Rawson lab who conducted pioneering work in isolating the *MBTPS2* gene. By utilizing two previously established cell lines, the M19 mutagenized Chinese hamster ovary (CHO) that is auxotrophic for cholesterol due to a defect in carrying out the site-2 cleavage to release the bHLHL-Zip domain of SREBPs from the membranes, and the HfT1M19(c) CHO that is a revertant cell line of the M19 CHO, as a recipient for complementation cloning, the *S2P* was isolated and characterized [10, 17]. The HfT1M19(c) CHO was transfected into the M19 CHO cells to generate complemented cells. This was repeated three times to eliminate extraneous human DNA and resulted in prototrophs that retained only a minute amount of human DNA with the rescue gene. Analysis of this gene revealed a protein of 519 amino acids whose sequence had a His-Glu-x-x-His (HExxH) motif which is a characteristic of zinc metalloproteases. Substitution of either of the two histidine residues or the glutamic acid blocked the site-2 cleavage of SREBPs, thus this protein was designated as S2P (site-2 protease) [10]. Further studies then revealed an additional motif, Leu-Asp-Gly (LDG), located ∼300 residues from the HExxH sequence that is equally essential for S2P activity [18].

The proteolytic function of S2P is further supported from the discovery of a family of related proteins in bacteria. These proteins are vital for the proteolysis of membrane-bound transcription factor needed for sporulation. For instance, the σ^k^ factor regulates gene expression in the mother cell after engulfment of the forespore. Cleavage of pro-σ^k^ and liberation of transcription factor require the membrane protein SpoIVFB, which this protein contains the HExxH motif [19]. In another bacterial S2P family member, YaeL in Escherichia coli, also harbors a HExxH motif that is essential for coordinating cell growth and cell division via intramembrane proteolysis of RseA, a factor activated in response to extracytoplasmic stress [20]. These studies have not only commenced a greater understanding of this protease but also has expanded the role of S2P beyond its proteolytic property to much broader roles.

### Activation transcription factor 6 (ATF6)

ATF6 activates the transcription of genes in response to the accumulation of unfolded or misfolded proteins in the ER [21]. As a type II ER TM protein, ATF6 contains a basic leucine zipper (bZip) domain at its NH_2_-terminal cytosolic domain, and a stress-sensing domain in the ER lumen [22]. Under resting conditions, ATF6 is retained in the ER through its association with the ER protein chaperone, BiP/GRP78 [23]. Upon accumulation of unfolded or misfolded proteins in the ER, ATF6 dissociates from BiP and migrates to the Golgi, where it is subjected to the sequential action of S1P and S2P, in a similar fashion to SREBPs [11]. The cleaved ATF6 cytosolic domain then migrates to the nucleus and induces the transcription of target genes, which encode ER stress proteins such as GRP78/BiP and XBP1 [24] (Fig. 1). Though ATF6 is widely known for its role in ER stress, there has also been reports of novel functions for ATF6 involving organogenesis and tissue homeostasis [25–27].

### Cyclic-AMP responsive element-binding protein 3 (CREB3)

The CREB3 family of transcription factors consist of five members—CREB3, CREB3L1, CREB3L2, CREB3L3, and CREB3L4. Like SREBPs and ATF6, all members of this family harbor a single TM helix with an NH_2_-terminal cytosolic domain resembling a transcription factor of the bZIP family and undergo proteolytic cleavage by S1P and S2P. They have been reported to have major roles in development, however, other roles have been identified such as metabolism, secretion of signaling proteins, cell survival, differentiation and division, and tumorigenesis.

CREB3 or Luman was first within its family to be identified as an ER-bound transcription factor as a counterpart of the herpes simplex virus transcriptional activator VP16 that binds to the host cell factor regulator [28, 29]. It is now well recognized to be involved in ER stress and unfolded protein responses as well as having regulatory roles in a multitude of processes related to the maturation of dendritic cells [30], Golgi stress [31], signaling of the glucocorticoid receptor [32], migration and function of leukocytes [33, 34], and metastatic progression of breast cancer [35]. Following stimulation, CREB3 is transported to the Golgi to be sequentially cleaved by S1P and S2P. The released NH_2_-terminal fragment relocates to the nucleus to activate target transcription genes and proteins such as cAMP response element (CRE), ER stress response element II, and unfolded protein response element [36, 37]. The activation leads to a transient translational attenuation, a transcription initiation of ER-resident chaperone folding capacity, and degradation of accumulated unfolded proteins in the ER, preventing cellular damage and apoptotic cell death [36] (Fig. 1).

CREB3L1, originally known as OASIS, plays a critical role in bone development. In response to bone morphogenetic protein-2 (BMP-2), a cytokine required for bone formation and osteoblast differentiation, CREB3L1 is cleaved sequentially by S1P and S2P [38, 39]. The cleaved NH_2_-terminal fragment enters the nucleus to initiate the transcription of COL1A1 by binding to a CRE-like sequence in its promoter region. Its prominent role in bone formation was further supported by its contribution as a genetic cause of OI [40–42] (Fig. 1). Mutations in *CREB3L1* downregulated the expressions of genes encoding components of the COPII coat, Sec23A and Sec24, protein components responsible for the transport and secretion of cartilage matrix proteins from the rough ER to the Golgi apparatus [43].

CREB3L2 is widely expressed in various tissue and organs, however, is preferentially expressed in proliferating chondrocytes in the cartilage [39, 43]. During chondrocyte proliferation, CREB3L2 is cleaved by S1P and S2P at the Golgi. The NH_2_-terminal domain of CREB3L2 then enters the nucleus and stimulates the transcription of genes encoding SEC23A and SEC24 [43]. Transcription of these genes expands the COPII-coated vesicles to accommodate the bulky type II collagen, an important component during chondrogenesis and chondrocyte differentiation [44] (Fig. 1). Additionally, CREB3L2 is implicated to have a role promoting collagen synthesis in dermal fibroblast also via the Sec23A pathway [45].

CREB3L3, also named CREB-H, was originally isolated as a hepatocyte-specific bZIP transcription factor and has an important role in innate immunity [12, 46]. Following microbial infection, the production of pro-inflammatory cytokines such as interleukin-6 (IL-6) by leukocytes results in ER stress in hepatocytes, which in turn triggers the proteolytic cleavage of CREB3L3 by S1P and S2P [12]. The cleaved NH_2_-terminal nuclear form activates the transcription of C-reactive protein and other acute phase proteins in the liver to provide an early defense against microbial infection [12, 47] (Fig. 1). On the contrary, a recent study found that the proteolytic cleavage of CREB3L3 in response to the production proinflammatory cytokines during myocardial ischemia causes a superimposed injury to myocardial cells [48]. Additionally, CREB3L3 has been implicated to have a role in ER-stress and proinflammatory cytokine TNFα-induced inhibition of osteogenesis. Following the inducement of tunicamycin, a potent inducer of ER stress, and TNFα in both MC3T3-E1 cells, a murine preosteoblast cell line, and primary osteoblasts, cleaved CREB3L3 blunted the BMP-2-induced up-regulation of osteogenic markers runt-related transcription factor 2 (RUNX2), alkaline phosphatase (ALP), and osteocalcin. This suggests of a negatively regulating role of CREB3L3 in osteoblast differentiation and bone formation [49, 50]. Interestingly, this is an opposite effect of BMP-2 on CREB3L1 despite being closely related. In another study, CREB3L3 was identified to have a role in osteoclastogenesis. Differentiation of osteoclast precursor cells via receptor activator of NF-κB ligand (RANKL)-induced differentiation, led to the accumulation of activated CREB3L3 in the nucleus and the activation of inflammatory transcription factor nuclear factor of activated T cells (*NFATC1*), a prominent transcription factor for osteoclast differentiation, to induce osteoclast. Inhibition of ER stress, however, reduced the expression of osteoclast-related genes and CREB3L3 activation, and thereby prevented RANKL-induced bone destruction, suggesting the inactivation of ER stress CREB3L3-induced signaling pathways may be of therapeutic use for the treatment of osteoporosis [51].

CREB3L4, also known as androgen-induced bZIP protein (AIbZIP), is highly abundant in the prostate where it is involved in the chromatin organization during spermiogenesis [52–54]. Like other members of the CREB3 family, CREB3L4, contains a single TM helix with an NH_2_-terminal cytosolic domain resembling a transcription factor of the bZIP family. However, unlike its relatives, the requirement to be sequentially cleaved by S1P and S2P at the Golgi for its activation remains slightly unclear. In one study using the human prostate cancer cell line, LNCaP, it was demonstrated that treatment with brefeldin A, a pharmacological compound that collapses the ER and Golgi to a single compartment, levels of full-length CREB3L4 were reduced, indicating that CREB3L4 undergoes S1P and S2P cleavage at the Golgi [55]. In a more recent study, however, CREB3L4 was found to be localized to the Golgi apparatus as well as the ER, suggesting it did not undergo proteolytic cleavage by S1P and S2P in normal and ER-stress induced conditions in LNCaP cells [56]. The activation of CREB3L4 may therefore require additional components or stimuli to undergo proteolytic cleavage for its activation. Despite this, CREB3L4 has been linked to numerous cellular process in addition to spermiogenesis, such as, cellular differentiation into adipocytes [57] or the progression of breast carcinoma [58].

## MBTPS2 gene and protein structure

The primary function of MBTPS2, as aforementioned above, is to cleave its substrates to enable them to perform vital cellular functions. To accomplish this, MBTPS2 relies on both of its conserved catalytic site motifs, HExxH and LDG, that are not only hallmarks of MBTPS2 but also core to its activity. Illuminating this was the structural studies of MBTPS2, via sequence-based topology predictions or X-ray crystallography methods, which has provided a substantial amount of information regarding the fundamental structural requirements of MBTPS2 and how they conform to its function as a membrane-embedded protease.

**Fig. 2:**
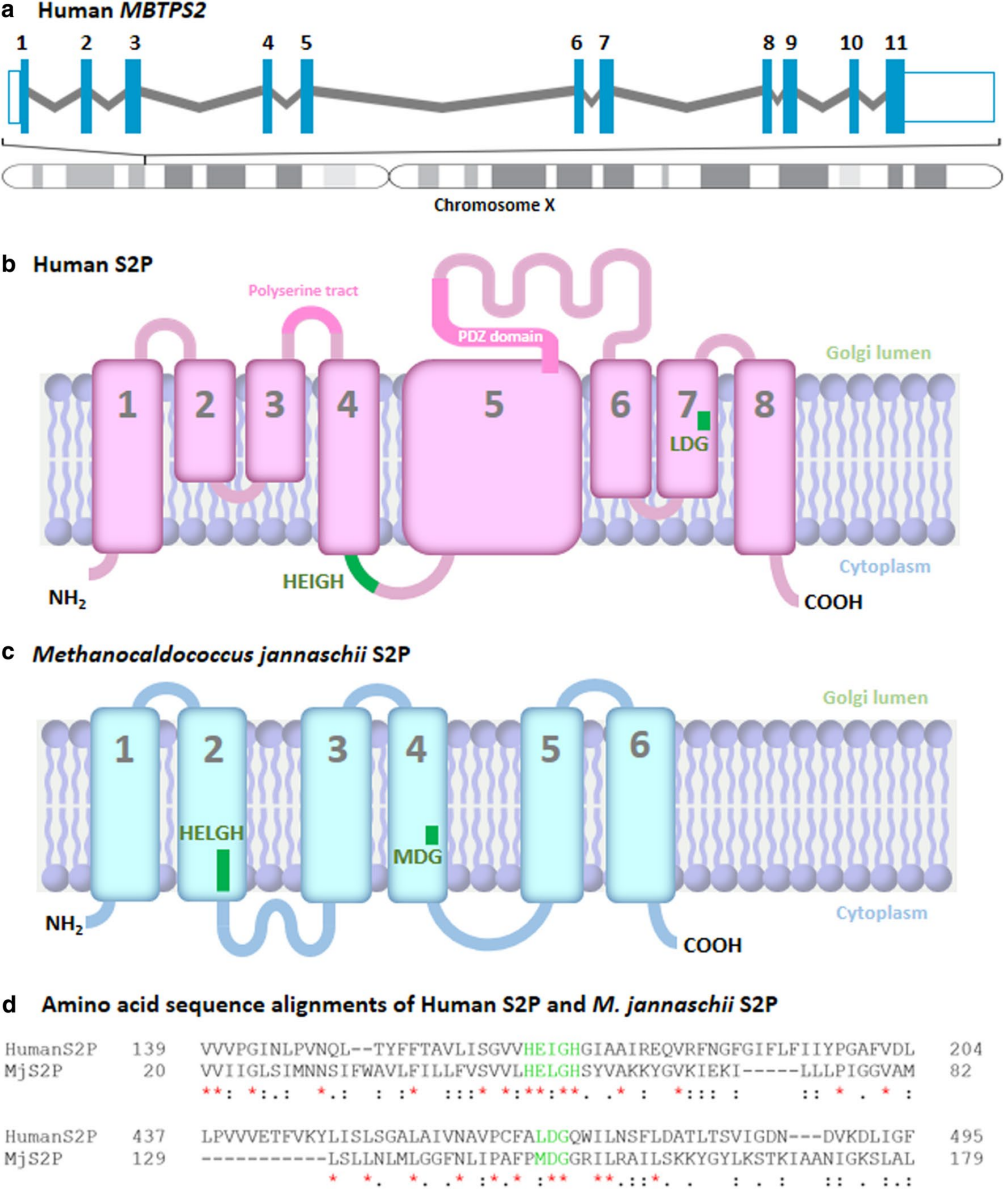
Organization of the MBTPS2 gene and the S2P protein structure. **a** Structure of human *MBTPS2* gene consists of 11 exons and is located on human chromosome X. **b** Predicted structure of human S2P protein contains eight transmembrane domains, a polyserine tract, a PDZ domain, and catalytic active motifs, HEIGH and LDG. **c** Membrane topology of MjS2P, adapted from Feng et al. [65]. **d** Amino sequence alignments of human S2P and MjS2P at the catalytic active motifs. Sequences were obtained from NCBI Protein Database, aligned using Clustal Omega Software [126], and cropped at the catalytic active motifs (green letters). An asterisk (*) indicates positions which have a single, fully conserved residue. A: (colon) indicates conservation between alignments of strongly similar properties. A. (period) indicates conservation between alignments of weakly similar properties

The *MBTPS2* gene spans greater than 4 kilobase pairs on human chromosome X and has 11 exons (Fig. 2a). The first glimpse of the MBTPS2 structure was deduced from a combination of protease protection and glycosylation studies, which revealed that it is 519 amino acids long and has a primary structure consisting of eight TM helices partitioned by four luminal loops and two cytoplasmic domains, an NH_2_- and a COOH-terminal [18] (Fig. 2b). Among the transmembrane domains, are the core segments of MBTPS2, the fourth (TM4) and the seventh (TM7) transmembrane that holds both of its conserved catalytic motifs, HExxH and LDG, respectively. The HExxH motif is a well-established signature of zinc metalloproteases that utilizes the two histidine residues to chelate a catalytic zinc ion and the glutamate residue to activate a water molecule for peptide bond hydrolysis. The LDG motif, another signature motif of metalloproteases, uses its aspartate residue to further facilitate the coordination of the zinc ion [18, 59]. Together these residues activate a zinc-bound molecule that initiates a nucleophilic attack at the scissile peptide bond of a substrate following S1P cleavage.

The luminal loops are made up of an uneven distribution of charged amino acids including lysine, arginine, glutamate, and aspartate [18]. The first luminal loop other than its hydrophilicity is considered unremarkable. The second loop is made up of 76% serine residues, though the importance of this run of serine residues is also yet to be defined. The third loop, which is the longest of the loops, is made up of 188 residues and contains a PDZ domain with a cysteine-rich insert, charged and polar amino acids, and a consensus sequence with homology to a glycosylated site. Protein glycosylation has not been suggested to be a necessary posttranslational modification for the activation or functioning of MBTPS2, unlike MBTPS1 that requires cleavage of its inhibitory portion prior to its activation [60]. Analysis of the PDZ domain, on the other hand, has been suggested to be essential in the recognition and/or binding of a newly synthesized COOH-terminus end of substrates following its cleavage by S1P [61]. Thus, the presence of a PDZ domain may be a potential explanation why a substrate must undergo cleavage by S1P prior to being cleavage by S2P [62]. PDZ domains are in fact present on the COOH-terminal of bacterial S2P homologs and deemed essential in substrate recognition. As an example, in Mycobacterium tuberculosis, removal of the PDZ binding domain of Rip1 (S2P) via truncation of the COOH-terminal, prevented the binding of the Rip1 to PDZ-interacting protease regulator 1. Thus, the PDZ binding domain acts as a substrate-specific adaptor protein that tethers the substrate to S2P for it to be cleaved and released in the cytosol [63]. The use of the PDZ domain as an a substate-specific adapter protein by the human S2P has yet to be demonstrated and is an area for discovery. The fourth luminal loop, shortest of the loops, bridges the last TM helix before the protein terminates at its COOH-terminal (Fig. 2b).

This predicted architectural view of the MBTPS2 structure has since been confirmed by sequence homologs studies [61, 64] and astonishingly by the first S2P atomic-resolution crystal structure obtained from an archaebacterium S2P (Methanocaldococcus jannaschii S2P (MjS2P)) [65] (Fig. 2c). Though the S2P crystal structure was obtained from the archaeal S2P homolog, there are striking parallels between the human S2P predicted sequence topology and the crystal structure of MjS2P (Fig. 2d). First, the placement of the two catalytic active sites at the cytosolic side of the membrane. Interestingly, this orientation complements well with the location of where SREBPs are cleaved which is near the cytosolic portion of the membrane [8, 66]. Second, the geometry of the active-site residues, the placement of the catalytic dyad, His-His and Asp for its catalytic activity indeed coordinate with each other and are spatially arrange in a manner similar to that seen with other HExxH-containing metalloprotease, such as thermolysin [67]. The only difference is, however, the MjS2P crystal structure suggests a six TM helix model, we speculate this difference is due to the human S2P being longer than the MjS2P.

The MjS2P crystal structure for the first time has given access to unprecedented structural and mechanistic insights of S2P at an atomic-level, providing answers to challenging questions and novel insights. The longest-standing conundrum of S2P proteolysis, as a matter of fact for most intramembrane proteases, has been the question of how a water-requiring proteolytic reaction can occur in a lipid membrane. The structure demonstrated the existence of a polar channel at the COOH-terminal which allows water entry to the active sites of S2P. Additionally, the inner surface of the polar channel is lined with several polar groups and charged amino acids including backbone carbonyls, and acidic and basic side chains, providing an aqueous microenvironment required for proteolysis. The structure also confirmed that proteolysis does indeed takes place within the plane of the membrane as the residues of the catalytic active sites were found to reside beneath the surface of the membrane. Another remarkable insight gained are hints of a ‘lateral gating mechanism’ that regulates substrate access to the active catalytic sites. This involves the pushing of TM1 and TM6 away from each other in a ‘double door opening’ fashion that exposes the S2P active sites. This translocation of the helices generates a crevice as wide as the length S2P to sufficiently accommodate the substrate [65]. Interestingly, this mechansim is similar to those established from the crystal structures of other membrane proteases such as rhomboids [68]. Thus, this allows us to begin to draw a picture of the core catalytic mechanisms of MBTPS2 that may serve as a conceptional framework for a deeper understanding of the machinery for its proteolytic activity. Nonetheless, this mechanism was inferred from the archaeal S2P homolog, thus it remains unclear if this gating mechanism will be of relevance for human MBTPS2 and it is unlikely to be fully resolved until complexes of S2P bound to a substate can be crystallized and their structure determined.

The successful resolving of the MjS2P structure may have faded former doubts of the proteolysis action of MBTPS2 but have also posed further questions. One particular aspect is that it has not been identified yet how substrates unfold or unwind to allow the catalytic sites of MBTPS2 to gain access to its scissile peptide bond. The process of cleavage of SREBPs may be utilized as a template: following S1P cleavage of SREBPs at their luminal loop, both of its TM segments separate from one another, causing it to partially unfold and its transcriptional domain easily accessed by S2P [59]. For the other substrates of MBTPS2, such as ATF6 and CREB3, their precise mechanism of cleavage has not been fully understood, thus it can only be speculated that these substrates must also be unfolded to allow its scissile peptide bond to be exposed to MBTPS2 catalytic sites. Another area that we have yet a real insight into is how MBTPS2 substrates are defined or selected. Initial mutational studies of SREBPs revealed two sequences that were critical for S2P cleavage [59]. The first, Asp-Arg-Ser-Arg (DRSR), which immediately precedes the first TM segment of SREBP-1a and SREBP-2. Changes to the DRSR sequence via substitutions or deletions severely disrupted the cleavage of S2P for SREBP-1a but did not have the same effect on SREBP-2. The second sequence important for S2P cleavage is Asn-Pro (NP) which sits within the first TM segment of SREBP-2. Changes to each residue of the NP sequence resulted in a partial reduction of S2P cleavage and changes to the both of sequence simultaneously, completely abolished the cleavage of SREBP-2 by S2P. Interestingly, translocating the NP sequence 5 residues NH_2_-terminally did not alter the position of cleavage nor impact its cleavage by S2P [59]. This may be suggestive of a recognition sequence, as it is not spatially regulated. However, it was not determined if cleavage at an altered position had any down-stream effects, perhaps at a transcriptional level. Similarly, ATF6 has a Asn-Tyr-Gly-Pro (NYGP) sequence within in its TM domain that is important for S2P cleavage, as substitution to both of the NP residues completely abolished cleavage [11]. The presence of the NP sequence in SREBP-2 and ATF6 is an exciting observation of a ‘specific recognition sequence’ that can dictate substate specificity of MBTPS2, rather than just a sequence cleavage, however, does require deeper analysis. The PDZ domain on MBTPS2 COOH-terminal may be an alternative route of a regulative mechanism, particularly the observations that certain bacteria S2P rely on its PDZ binding motif as an adapter protein for substrate specificity. The MjS2P, however, did not have any domain or motif inserts of such between its TMS or luminal loop, instead, had a rather noticeable cavity in its structure and we are tempted to speculate that this would be the region where the PDZ binding motif would sit.

Overall, these emerging proposals are plausible, but there is more to be understood about the details of S2P substrate entry, its catalytic mechanism, and specifications of what makes an S2P substrate. Perhaps, genome editing approaches such as CRISPR-Cas9 of known substates combined with structures of S2P in complex with its substates, will aid in resolving this. Implications of these will not only be a subject of interest on mechanistical and theoretical levels but has major connotations in understanding how MBTPS2 functions in various biological contexts, diseases, and the possibility of utilizing MBTPS2 and its substrates as a therapeutic target.

## MBTPS2 associated diseases

Given the significance in cholesterol homeostasis and ER stress responses, and the many substrates and cellular functions that rely on the proteolytic activity of MBTPS2, it is not surprising that mutations leading to MBTPS2 deficiency can lead to multiple diseases. To date, mutations in the *MBTPS2* gene have been reported to cause debilitating disorders including IFAP syndrome with or without BRESHECK, KFSD, Olmsted and most recently, OI.

### Ichthyosis follicularis, alopecia and photophobia (IFAP) syndrome with or without BRESHECK

**Fig. 3:**
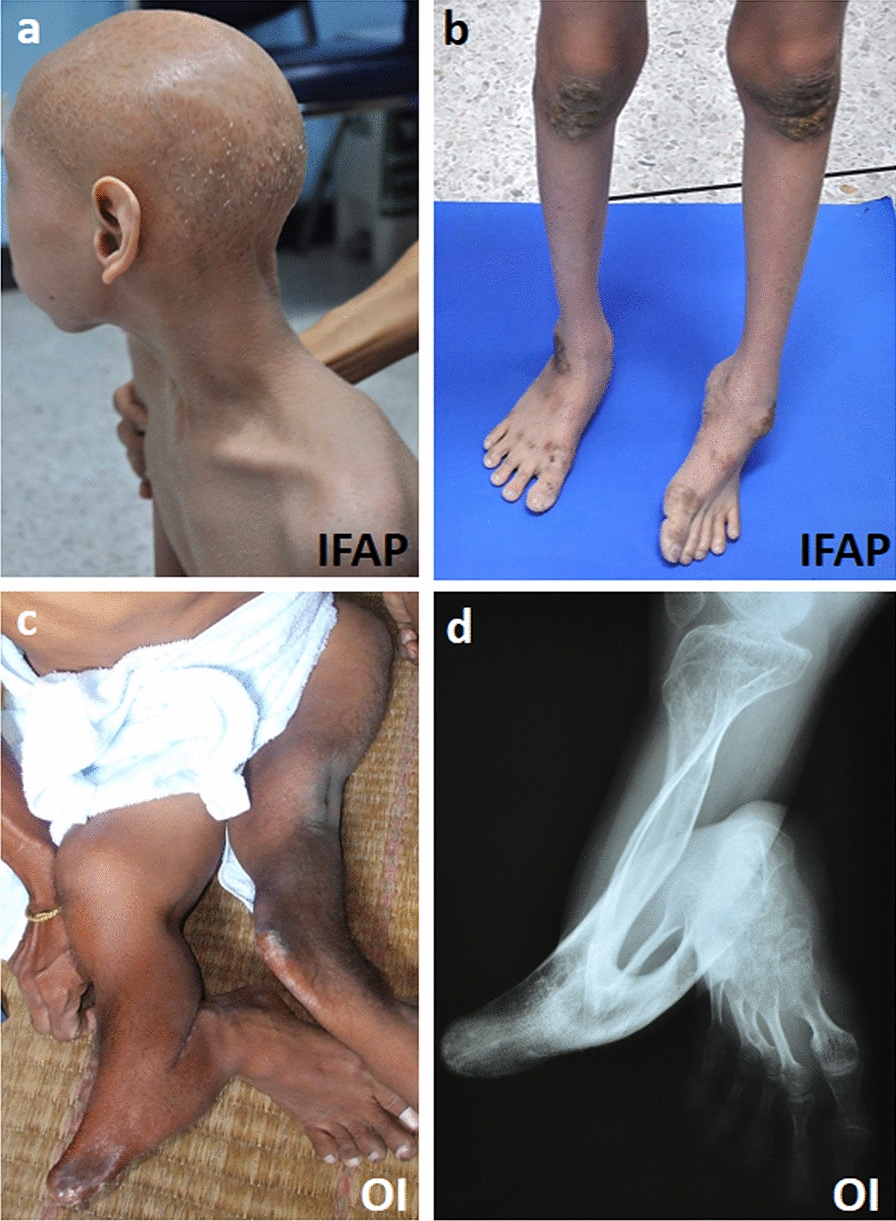
Clinical and radiographic images of patients with IFAP or OI caused by *MBTPS2* mutations. **a**, **b** A patient with IFAP harboring the p.R429H mutation in *MBTPS2* exhibited alopecia, ichthyotic scaling, scaly hyperkeratotic plaques over scalp, hyperkeratosis, and dystrophic nails **c**,** d** A patient with OI harboring p.N459S mutation in *MBTPS2* exhibited a severely anterior angulation of legs

IFAP syndrome (MIM #308205) is an extremely rare congenital disorder with only about 60 cases reported worldwide since it was first identified by McLeod in 1909 [69]. The syndrome is inherited in an X-linked recessive fashion and primarily affects males, however, there have been reports of autosomal-dominantly inherited IFAP cases in females [70–72]. Female carriers have also been reported to display symptoms, though the trait may be nonpenetrant or are mild such as follicular ichthyosis, mild atrophoderma, hairlessness, and hypohidrosis in a linear mosaic pattern [73, 74]. The phenotypic spectrum of this syndrome in males is variable, ranging from mild to severe, but all affected individuals display the peculiar triad of follicular ichthyosis, nonscarring generalized absence of hair and abnormal sensitivity to light [75]. Ichthyosis follicularis, a hallmark feature of this syndrome, arises as widespread non-inflammatory thorn-like follicular keratosis, which many occur at the scalp and extensor extremities (Fig. 3a, b). Additional cutaneous defects may include hyperkeratotic psoriasiform plaques, lamellar scaling, angular cheilitis, periungual inflammation and dystrophic nails. Noncicatricial alopecia, the most prominent manifestation, is the loss of hair at the scalp, eyebrows and eyelashes or a complete body hair loss. Superficial corneal ulceration and vascularization lead to progressive scaring of the cornea and possibly cause a defect in Bowman membrane, which underlies photophobia, the third defining characteristic of the triad. Further ocular manifestations include corneal erosions and scarring, chronic tearing, nystagmus, myopia, and atopic keratoconjunctivitis. The anterior chamber, lens, and ocular fundus are not affected. Further manifestations may occur together with the triad such as intellectual disability, seizures, hypotonia, short stature, frontal bossing, choanal atresia, recurring infections, and intestinal irregularities as well as renal, cardiac, and vertebral anomalies [76]. In extreme cases, an assortment of manifestations can be presented in affected individuals including brain anomalies, intellectual disability, ectodermal dysplasia, skeletal deformities, ear or eye anomalies, and renal anomalies or small kidneys, with or without Hirschsprung disease and cleft palate or cryptorchidism (BRESEK or BRESHECK) [77].

**Fig. 4:**
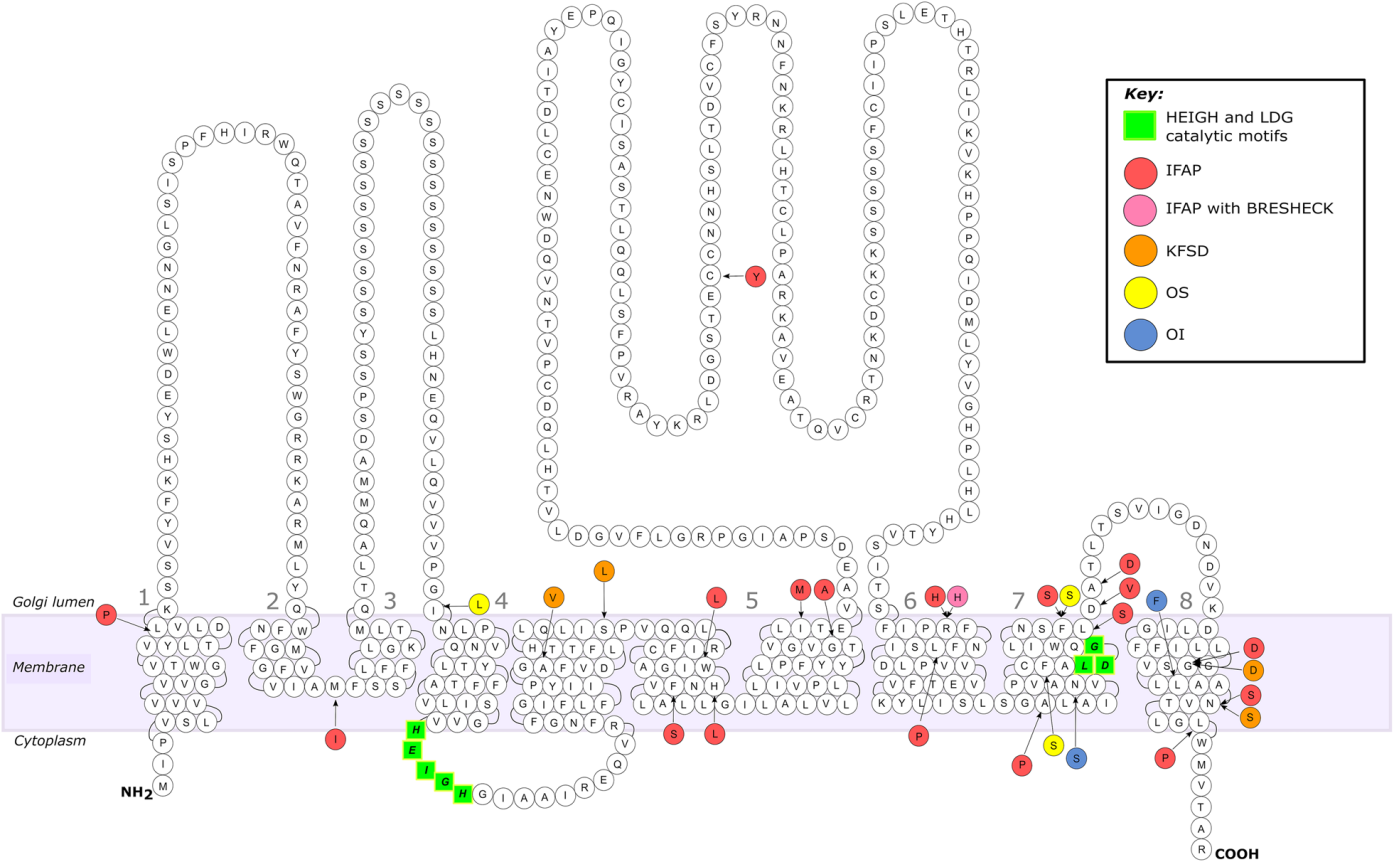
Reported pathogenic *MBTPS2* variants. MBTPS2 protein structure contains eight transmembrane domains (TM 1 to TM 8) spanning the Golgi membrane. The NH2-terminal, COOH-terminal and the loop linking TM 4 and 5 are cytoplasmic, whilst the four loops are luminal. The schematic diagram is adapted from Bornholdt et al., 2013. Green highlighted amino acids are the catalytic active motifs, HEIGH and LDG. Pathogenic variants with the substituted amino acids are indicated by arrows and colored circles. Red amino acids are substitutions that cause IFAP syndrome. Pink amino acids are substitutions that cause IFAP with BRESHECK. Orange amino acids are substitutions that cause KFSD. Yellow amino acids are substitutions that cause OS. Blue amino acids are substitutions that cause OI

Though identified over a century ago in 1909, the genetic basis of this rare disorder was only uncovered in 2009 [78]. By linkage analysis and sequencing of the candidate genes, five unrelated affected males with the IFAP syndrome were each found to harbor a missense mutation in *MBTPS2* (Fig. 4, Table 1). The five identified missense mutations, p.R429H, p.H227L, p.M87I, p.W226L, and p.F475S, resulted in the abolishment of the proteolytic activity of S2P, with the extent of S2P proteolytic activity loss specific to each mutation. This was determined by the analyses of cell viability in M19 CHO cells bearing the different detected missense mutations. Under sterol-depleted conditions, the five mutants displayed varying degrees of poor cell growth compared to M19 cells transfected with wild-type MBTPS2. Most strikingly was the p.R429H mutant that had almost no detectable cell growth and the lowest residual proteolytic activity. Interestingly, the affected male harboring this mutation also had the most prominent IFAP phenotypes, suggestive of a correlation between clinical severity and the extent of impairment in S2P proteolytic function as a consequence of the mutation [78]. This mutation lies within the domain of the COOH-terminal located near the LDG motif at TM 7, one of the two proteolytic active sites of S2P, further hinting at a relationship between proteolytic function and clinical severity.

**Table 1:** Disorders and main clinical features associated with *MBTPS2* mutations

Disorders	Main clinical features	Mutation	Amino acid change
IFAP	Ichthyosis follicularis, alopecia, photophobia, non-inflammatory thorn-like follicular keratosis, hyperkeratosis, dystrophic nails, ectopic keratoconjunctivitis, corneal scars, corneal erosion, and neovascularization	c.71T > C	p.L24P
		c.261G > A	p.M87I
		c.667G > T	p.W226L
		c.680A > T	p.H227L
		c.686T > C	p.F229S
		c.758G > C	p.G253A
		c.774C > G	p.I258M
		c.1001G > A	p.C334Y
		c.1286G > A	p.R429H
		c.1360G > C	p.A454P
		c.1424T > C	p.F475S
		c.1427T > C	p.L476S
		c.1430A > T	p.D477V
		c.1433C > A	p.A478D
		c.1499G > A	p.G500D
		c.1523A > G	p.N508S
		c.1538T > C	p.L513P
		c.671-9T > G	p.I225Lfs*25
IFAP with BRESHECK	Ichthyosis follicularis, atrichia, photophobia, with brain anomalies, intellectual disability, ectodermal dysplasia, skeletal malformations, Hirschsprung disease, ear deformity and deafness, eye hypoplasia, cleft palate, cryptorchidism, and kidney dysplasia/hypoplasia (BRESHECK)	c.1286G > A	p.R429H
KFSD	Diffuse follicular hyperkeratosis, progressive cicatricial alopecia of the scalp, eyebrows, and eyelashes, photophobia, blepharitis/conjunctivitis, and corneal dystrophy	c.599C > T	p.A200V
		c.1499G > A	p.G500D
		c.1523A > G	p.N508S
		c.638C > T	p.S213L
OS	Periorificial keratotic plaques, bilateral palmoplantar transgredient keratoderma, diffuse alopecia, leukokeratosis of oral mucosa, onychodystrophy, hyperkeratotic linear streaks, follicular keratosis, and constriction of digits	c.671-9T > G	p.I225Lfs*25
		c.1391T > C	p.F464S
		c.1424T > C	p.F475S
OI Type XIX	Low bone mass, progressive bone deformities with increased fracture frequencies, craniofacial abnormalities, scoliosis, atraumatic subluxations, dentinogenesis imperfecta, hearing impairments, blue sclerae, and lung abnormalities	c.1376A > G	p.N459S
		c.1515G > C	p.L505F

*IFAP* Ichthyosis follicularis, atrichia and photophobia syndrome, *OI* Osteogenesis imperfecta, *KFSD* Keratosis follicularis spinulosa decalvan, *OS* Olmsted syndrome

Regarding genotype–phenotype correlation, it appears that mutations lying within in or close to TM 5 and 8 contribute to severe manifestations, while mutations closer to the NH_2_- or COOH-terminals result in milder forms of IFAP. This is not only evident for the p.R429H mutation but also for the p.L433P, p. A454P, p.F475S, p.L476S, p.D477V, p.A478D, and p.G500D mutations that are as well adjacent to the LDG motif and led to clinically severe IFAP phenotypes [78–81]. Additionally, mutations within the TM 5, p.F229S, p.W226L, p.H227L, p.G253A, and p.I258M are also associated with considerably severe IFAP phenotypes. Interestingly, a mutation was identified at the large luminal loop between TM 6 and 7 (p.C334Y), which the affected male displayed classic clinical features of IFAP along with psoriasiform skin plaques, nail dystrophy, facial dysmorphism, intellectual disability, severe skeletal abnormalities, and chorea-like movement. Though the IFAP phenotypes of this mutation were not severe, it may suggest that a mutation at this site may be particular to severe skeletal anomalies. However, this remains to be clarified [82]. A patient with a p.L24P mutation, the most-proximal mutation close to the NH_2_-terminal end on TM 1 had mild IFAP phenotypes but also had global developmental delay [83]. In two other patients, one harboring the p.M87I mutation, the second most-proximal mutation on TM 2, and the p.L513P mutation, the most COOH-terminal encountered mutation on TM 8, resulted in milder phenotypes of IFAP [78, 79] (Fig. 4). Nonetheless, it could be contended that no specific phenotype or genotype correlation can be formed from mutation positions. In one study it was reported that a patient carrying the p.R429H mutation displayed mild phenotypes of IFAP, in contrast to what was previously observed, the patient however, did experience neurological abnormalities such as retarded psychomotor development and seizures [78, 84]. Additionally, another patient was not affected by the triad of IFAP but by BRESHECK with atrichia and photophobia [85]. Yet, another patient severely manifested the cardinal triad of IFAP with an additional five features of BRESHECK [77]. Precaution, therefore, must be taken when making predictions of clinical outcome from a specific mutation.

Initially, BRESEK or BRESHECK was considered as a distinct entity that was termed ‘BRESEK or BRESHECK syndrome’ by Reish et al. (1997); however, as majority of its symptoms are often presented along side the IFAP triad, particularily Hirschsprung disease in severe cases of IFAP, IFAP, and BRESHECK have been classified within the same entity as ‘IFAP with or without BRESHECK (MIM #308205) [77–79, 85, 86]. In essence, prior to the coining of the ‘BRESEK or BRESHECK syndrome’ Martino et al. (1992) reported a male patient that was affected by the IFAP triad and additional symptoms that were very much alike to those of the BRESHECK syndrome, including short stature, intellectual disability, seizures, hypohidrosis, enamel dysplasia, congenital aganglionic megacolon, inguinal hernia, vertebral and renal anomalies, however the genetic basis underlying IFAP and the additional phenotypes of this patient was not determined [76].

### Keratosis follicularis spinulosa decalvans

Keratosis follicularis spinulosa decalvans (KFSD, MIM #308800) is a rare hereditary disorder of keratinization recognized by widespread hyperkeratotic follicular papules [87]. First described in 1926, the disorder is often presented at infancy or early childhood with an X-linked pattern of inheritance, though sporadic cases or cases inherited in an autosomal dominant fashion have been reported [88–92]. Some affected individuals exhibit extensive keratosis pilaris‐like papules, as well as facial erythema, hypotrichosis, and cicatricial alopecia of the scalp, eyebrows, and eyelashes. Extracutaneous features include photophobia, keratitis, blepharitis, and enamel hypoplasia. As the phenotypes of this disorder considerably simulates IFAP, KFSD is sometimes considered as a ‘milder form of IFAP, however, this disorder can be distinguished from IFAP via the nature of alopecia, which is progressive with variable degrees of inflammatory change leading to scarring in KFSD.

Mutations in *MBTPS2* have been identified as one of the causative genes of KFSD. Alike mutations leading to the IFAP triad, mutations underlying KFSD lie within the TM domains of MBTPS2. In three unrelated families, a missense mutation in *MBTPS2* (p.N508S) was identified in affected males displaying mild phenotypes [87]. Following this, two independent studies reported a Chinese and a Swedish family in which the same mutation segregated with mild phenotypes of KFSD [79, 92]. The position of this recurrent mutation was mapped to be at the COOH-terminal end of MBTPS2 and a genotype–phenotype effect specific to *MBTPS2* mutation was speculated (Fig. 4). Indeed, cell viability assays of M19 CHO cells possessing the p.N508S mutation showed a 50% reduced growth rate in sterol deprived conditions compared to wild-type, though, in comparison to the mutations underlying severe phenotypes of IFAP, the p.N508S mutation had a higher survival rate [78, 79, 87]. Not only does this strengthens the notion that mutations located far away from the active proteolytic site do not lead to severe phenotypes but may also suggest that localization of mutations can be utilized as a marker of clinical severity [78]. Subsequently, additional reports of missense *MBTPS2* mutations have been linked to mild phenotypes of KFSD and abide to the genotype to phenotype pattern [79, 93, 94].

### Olmsted syndrome

Olmsted syndrome (OS, MIM #614594) is an extremely rare keratinization genetic disorder classically marked by the combination of bilateral mutilating palmoplantar keratoderma, and periorificial hyperkeratotic plaques [95]. The disease normally presents at birth or early childhood, however, onset in adults have been reported [96]. Diagnosis of OS generally relies on the clinical presentation of bilateral mutilating palmoplantar keratoderma and periorificial hyperkeratotic plaques, though there is a high variability in its phenotypic spectrum by the presence of accompanied features. These include corneal opacities, diffuse alopecia, digital constriction rings, nail dystrophy, high-tone hearing impairment, infections, hyperkeratotic linear streaks at the elbows, knees, axillae, and antecubital fossae, and squamous cell carcinomas [97, 98]. As such, diagnosis is often difficult and confusing as majority of its phenotypes overlap with IFAP. To complicate things further, *MBTPS2* mutations via an X-linked mode of inheritance has recently become alight to cause OS, in addition to the classical OS-causative gene, transient receptor potential cation channel, subfamily V (TRPV3) [74, 99–104].

Among the 106 cases of OS, three of these are to date reported to be caused by mutations in *MBTPS2*. First of the three is a missense mutation (p.F464S) harbored by two males in an Iranian pedigree [99, 105]. This missense mutation is located on TM 7 and only two codons away from the LDG catalytic center of MBTPS2. Rather close to the LDG motif, severe OS manifestations were expected. Accordingly, the two probands severely displayed all the features of OS, with alopecia universalis, painful hyperkeratotic lesions, severe periorificial plaques, and severely dystrophic nails with a fork-like appearance and yellow in color. In another pedigree, a c.671-9 T > G intronic mutation was present in a Chinese proband that exhibited not only features of OS but also the IFAP triad with short stature, inguinal hernia, palmoplantar, periorificial keratoderma, and pachyonychia [74]. Interestingly, this mutation was previously reported in two IFAP patients that also exhibited hernia, short stature, and thickened dystrophic nails, but no symptoms of OS [78]. By an in vitro mini gene assay and reverse transcription PCR, this mutation disrupted the intronic splicing enhancer, resulting in the skipping of exon 6 for mRNA transcription and a frameshift that prematurely terminated MBTPS2 (p.I225Lfs*25) [74, 78]. This diversifies the heterogeneity of *MBTPS2* mutations to not only consist of mutations on TM domains but also ones at the exon–intron boundaries of *MBTPS2*. The third mutation is another recurrent mutation (p.F475S) that was reported in two Lebanese brothers that displayed clinical features for both OS and IFAP [104] (Fig. 4). This missense mutation had previously been documented in an Argentinian male and two Lebanese brothers with severe manifestations of IFAP with hyperkeratotic psoriasis‐like lesions and plantar keratoderma, respectively, amongst other features [78, 79]; however, there were no mentions of OS. Whilst, in another report by Nemer et al. (2017), two affected Lebanese brothers displayed phenotypes of both IFAP and OS with development delays, recurrent seizures, osteoporosis, and renal insufficiency [104]. These reports together underscore the challenge in reaching at a consensus for diagnosis when OS cases with IFAP manifestations or vice versa are presented simultaneously. As a result, there has been controversy over whether X-linked OS should exist as an independent syndrome or just as a severe form of IFAP [74, 106]. The reason for debate arose from the observation that, patients displaying clinical features of OS occasionally also show IFAP phenotypes. This contrasts with the onset of OS due to *TRPV3* mutations which affected individuals only display classical OS phenotypes without any IFAP features. Whether the nomenclature of OS should be incorporated with IFAP in a similar manner that the BRESHECK syndrome was grouped as ‘IFAP with or without BRESHECK’ requires further considerations.

### Osteogenesis imperfecta (OI)

Osteogenesis Imperfecta (OI) or brittle bone disease is a congenital heterogenous skeletal deformity disorder that affects approximately 1 in 10,000–20,000 births worldwide [107]. Individuals with OI display a marked skeletal phenotype with a broad clinical spectrum of severities that varies from low bone mass to progressive bone deformities with increased fracture frequencies and perinatal lethality. Additionally, OI individuals may exhibit an array of associated secondary features including craniofacial abnormalities, scoliosis, atraumatic subluxations, and dentinogenesis imperfecta as well as extra-skeletal manifestations including hearing impairments, blue sclerae, and lung abnormalities [108, 109].

Typically, OI is most often found to be caused by autosomal dominant mutations in either of the genes that encode for collagen type 1 alpha chains, *COL1A1* or *COL1A2*, causing an alteration in the structure or function of collagen type 1, the most abundant protein of bone extracellular matrix [108, 110–112]. Mutations in genes involved in posttranslational modification of collagen (*CRTAP, PPIB, LEPRE1/P3H*1) [113–115], folding (*SERPINH1, FKBP10*) [13, 116], intracellular trafficking (*SEC24D*) [117], and extracellular processing (*BMP1*) [118, 119] have also been described to cause OI as a result of autosomal recessive inheritance. Over the past decade, however, the advent of high-resolution sequencing technologies such as next-generation sequencing (NGS) and the increased application of whole-exome sequencing has greatly widened the horizon of the genetic contributions to OI. These approaches have led to the identification of new OI-causing genes and novel pathogenic variants that are not classically associated with collagen metabolism and can occur via distinct inheritance patterns, such as the first X-linked recessive form of OI (OI type XIX, OI19, MIM #301014), caused by missense mutations in *MBTPS2* [3, 120, 121] (Fig. 3c, d). In two independent pedigrees with moderate to severe OI, linkage analysis and NGS revealed two novel *MBTPS2* missense mutations. In both pedigrees, the resulting substitutions (p.N459S and p.L505F) were found to occur within or adjacent to the zinc ion coordinating site required for enzymatic activity (Fig. 4). Expression levels of mutant S2P were stable, however, processing of the RIP substrates ATF6, CREB3L3, and SREBPs were impaired, resulting in decreased expression levels of several genes critical for osteoblast differentiation and extracellular matrix synthesis.

The underlying molecular mechanism for the phenotypic differences for these very distinctive disorders is currently unclear but may potentially be revealed by an in‐depth characterization of intracellular signaling differences associated with the skin disorders or OI mutations using stem cell-based disease models such as induced pluripotent stem cells (iPSCs) and/or animal models [122]. Any insights gained from these studies will further our understanding of the MBTPS2 properties in disease, and thereby assist in the possibility of utilizing MBTPS2 as a therapeutic target for the treatment of its associated skin disorders and OI. Interestingly, it has been demonstrated that Nelfinavir, an HIV protease inhibitor, was able to induce apoptosis and growth arrest of both liposarcoma and castration‐resistant prostate cancer, through the inhibition of S2P activity [123–125], suggesting a potential role of utilizing S2P as a therapeutic target for certain malignancies.

## Conclusions

Since the serendipitous discovery of MBTPS2 in 1997, tremendous strides have inarguably been made in understanding the role of this once heretical protease. It has transition S2P from just a component in the cleavage machinery of SREBPs to now a protease that is widely regarded as a critical regulator of serval cellular processes in health and disease. The biochemical and structural biology studies have offered spectacular insights into the mechanisms and functions of S2P, disposing the longstanding doubt of whether its proteolytic action via hydrolysis of peptide bonds can occur in the membrane, provided a conceptual platform in appreciating the broader functions of S2P and revealed a plethora of substrates that are entirely dependent on S2P for their downstream target pathways. Another notable insight gained is the identification of both of the catalytic active sites of S2P that is not only crucial to its proteolysis, but also seemingly an essential aspect in the pathogenesis of the disorders resulting from *MBTPS2* mutations in which several studies have revealed a prominent concordance in the molecular and biochemical properties of the mutations to the catalytic sites. What is also rather striking is the very distinctive disorders that result from the variants of *MBTPS2*, which may be suggestive of MBTPS2 having pleiotropic properties. Additionally, the disorders that so far have been reported arise from mutations that all lead to loss of function of MBTPS2, an area that has not been delved into are effects or disorders of variants that lead to the hyperactivity of MBTPS2. Nonetheless, the challenge lies in deciphering the complex pathomechanism underlying the associated distinctive disorders. Investing efforts into these important, albeit challenging, technical areas will most definitely accelerate our understanding of MBTPS2 in pathophysiological conditions even further. Looking into the future we hope that many of the fundamental remaining questions about MBTPS2 will be addressed and look forward to the exciting advances in both the mechanistic features of MBTPS2 functions and to utilize it as a therapeutic target.

## Data Availability

All data generated or analyzed during this study are included in this published article.

## Abbreviations

ATF6: Activating transcription factor 6
bHLHL-Zip: Basic-helix-loop-helix-leucine zipper
BMP-2: Bone morphogenetic protein-2
BRESHECK: Brain anomalies, intellectual disability, ectodermal dysplasia, skeletal deformities, ear or eye anomalies, and renal anomalies or small kidneys, with or without Hirschsprung disease and cleft palate or cryptorchidism
CHO: Chinese hamster ovary
COOH terminal: Carboxyl terminal
CREB3L1: Cyclic AMP-responsive element-binding protein 3-like protein 1
CREB3L2: Cyclic AMP-responsive element-binding protein 3-like protein 2
CREB3L3: Cyclic AMP-responsive element-binding protein 3-like protein 3
CREB3L4: Cyclic AMP-responsive element-binding protein 3-like protein 3
IFAP: Ichthyosis Follicularis, Atrichia and Photophobia syndrome
KFSD: Keratosis follicularis spinulosa decalvans
MBTPS1: Membrane-bound transcription factor protease, site-1
MBTPS2: Membrane-bound transcription factor protease, site-2
MjS2P: Methanocaldococcus jannaschii S2P
NH2-terminal: Amino-terminal
OI: Osteogenesis imperfecta
OS: Olmsted syndrome
RANKL: Receptor activator of NF-κB ligand
RIP: Regulated intramembrane proteolysis
S1P: Site-1 protease
S2P: Site-2 protease
SREBPs: Sterol regulator element binding proteins
TM: Transmembrane
